# mTOR co-targeting strategies for head and neck cancer therapy

**DOI:** 10.1007/s10555-017-9688-7

**Published:** 2017-08-18

**Authors:** Zhiyong Wang, Juan Callejas Valera, Xuefeng Zhao, Qianming Chen, J. Silvio Gutkind

**Affiliations:** 1 Moores Cancer Center, University of California San Diego, La Jolla, CA USA; 2 State Key Laboratory of Oral Diseases, National Clinical Research Center for Oral Diseases,West China Hospital of Stomatology, Sichuan University, Chengdu, Sichuan 610041 China

**Keywords:** mTOR, Head and neck cancer, Precision therapy, Immune oncology

## Abstract

Head and neck squamous cell carcinoma (HNSCC) is the sixth most common malignancy worldwide. There is an urgent need to develop effective therapeutic approaches to prevent and treat HNSCC. Recent deep sequencing of the HNSCC genomic landscape revealed a multiplicity and diversity of genetic alterations in this malignancy. Although a large variety of specific molecules were found altered in each individual tumor, they all participate in only a handful of driver signaling pathways. Among them, the PI3K/mTOR pathway is the most frequently activated, which plays a central role in cancer initiation and progression. In turn, targeting of mTOR may represent a precision therapeutic approach for HNSCC. Indeed, mTOR inhibition exerts potent anti-tumor activity in HNSCC experimental systems, and mTOR targeting clinical trials show encouraging results. However, advanced HNSCC patients may exhibit unpredictable drug resistance, and the analysis of its molecular basis suggests that co-targeting strategies may provide a more effective option. In addition, although counterintuitive, emerging evidence suggests that mTOR inhibition may enhance the anti-tumor immune response. These new findings raise the possibility that the combination of mTOR inhibitors and immune oncology agents may provide novel precision therapeutic options for HNSCC.

## Background

Head and neck squamous cell carcinoma (HNSCC) is the sixth most common malignancy worldwide. As a major public health concern, HNSCC arises in the oral cavity, larynx, and pharynx, affecting approximate 600,000 patients each year [1], only 40–50% of which will survive more than 5 years [2]. The leading risk factors include the use of tobacco, alcohol, and betel quid and areca nut chewing, while high-risk human papillomavirus (HPV) infection has emerged as a major risk factor, nowadays accounting for more than 20% of all HNSCC cases [3]. Currently, the main therapeutic modalities include surgery, radiation, and chemotherapy. However, these nonselective treatments may cause associated morbidity and mortality and usually have high systemic toxicities. Cetuximab, a monoclonal antibody-inhibiting EGFR, is the only cancer-targeting agent approved for HNSCC, although only ~10% of HNSCC patients respond to this agent and often for a short period of time [4, 5]. Two immune check point inhibitors targeting PD-1 have been recently approved by the FDA for HNSCC patients, albeit the rate of response is approximately 20%, lower than that of other malignancies, such as melanoma [6–14]. There is an urgent need to develop new effective strategies to prevent and treat HNSCC. Understanding the contribution of genomic alterations driving HNSCC initiation and progression may help explore novel precision therapeutic options.

## Genomic landscape in head and neck cancer

Deep sequencing approaches for the study of cancer genomes have recently revolutionized medical oncology [15]. By providing an unprecedented knowledge of the multiplicity and diversity of genomic and epigenetic alterations which underlie every individual cancer lesion, these approaches deepen the understanding of dysregulated signaling circuitries and molecular mechanisms driving cancer development. Based on these information, novel druggable targets for therapeutic interventions in various human malignancies have been revealed. Several recent reports [16–19] and a landmark study from the Cancer Genome Atlas (TCGA) Network [1] has provided a comprehensive genomic characterization of HNSCC, revealing hundreds of mutations in each HNSCC lesion. This complexity of genomic alterations makes it daunting to search for molecular events driving the development of cancer, especially to differentiate driver from passenger mutations, the latter having a minimal influence on tumor progression and/or therapeutic response. However, in-depth analysis of the HNSCC oncogenome suggests that despite the complexity of the distinct molecular alterations in individual lesions, they all fall within a limited number of dysregulated molecular pathways that may contribute to most HNSCC patients [1, 3].

Specifically, the most frequently identified alterations in HNSCC participate in biologic processes regulated by the *TP53* (71% mutated), *FAT1* (23% mutated and 5% deleted), *NOTCH1* (9% mutated and 66% signaling pathway alterations), *CASP8* (10% mutated), *CDKN2A* (22% mutated and 60% gene copy loss) genes, and *PIK3CA* (~20% mutated and 30% signaling pathway alterations) [3]. This reductionist approach based on comprehensive genomic profiling may be exploited to distinguish oncogenic signaling-related subgroups from unselected cancer cohorts and facilitate the identification of actionable therapeutic targets for HNSCC patients.

## Activation of PI3K-mTOR signaling pathway in head and neck cancer

**Fig. 1:**
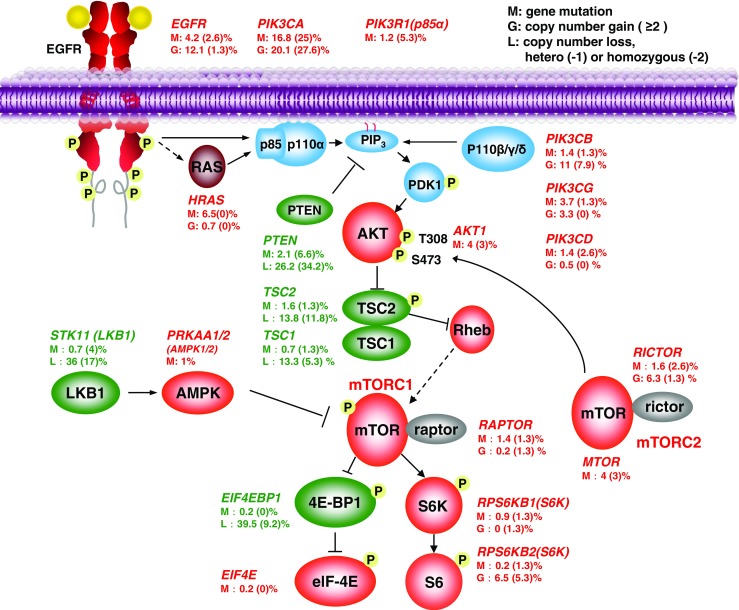
Frequent genetic alterations of PI3K/mTOR signaling pathway in HNSCC. Data was extracted from the HNSCC Cancer Genome Anatomy (TCGA) effort, including 428 HPV(−) and 76 HPV(+) HNSCC samples. Alterations identified in each key gene are shown, percentages outside and inside parentheses represent HPV(−) and HPV(+) samples, respectively. Red represents oncogene mutations and amplifications, and green represents tumor suppressor gene mutations and copy losses (copy loss refers to homozygous and heterozygous deletion of genes)

A more pathway-specific analysis of the HNSCC oncogenome suggests that most genomic alterations are involved in aberrant mitogenic signaling routes, including the PI3K, MAPK, and JAK/STAT pathways [17]. Remarkably, the PI3K-mTOR pathway is mutated in the highest percentage of the cases. In contrasts, MAPK and JAK/STAT pathways harbor mutations in less than 10% of the lesions. Specifically for PI3K, the in-depth analysis of TCGA data from 428 HPV− and 76 HPV+ HNSCC samples [20] revealed that *PIK3CA* is the highest mutated gene when considering all HNSCC cases (16.8%), and PI3K mutations (*PIK3CA*, *PIK3CB*, *PIK3CG*, and *PIK3CD*) are frequently mutated genes in 30% of the HPV+ lesions. Moreover, PI3K mutations are not the only genomic alterations causing the persistent activation of PI3K/AKT/mTOR pathway in HNSCC. Various genetic and epigenetic changes coordinate with PI3K mutations to sustain activation of this pathway in HNSCC (Fig. 1).

For instance, DNA copy number gain and messenger RNA (mRNA) overexpression of *PIK3CA* frequently occur in HNSCC (20 and 52%, respectively). Other PI3K isoforms and multiple PI3K regulatory subunits also have mutations and copy number gains (0.5–11%). Over 90% of HNSCC lesions overexpressed the epidermal growth factor receptor (EGFR), which is upstream of PI3K/AKT signaling, a major driver of epithelial cell proliferation. And a low frequency of HNSCC cases has mutations in *AKT2* and *mTOR* or its regulatory subunits, *RICTOR* and *RAPTOR*. A network-based analysis of the HNSCC oncogene revealed that a high percentage of lesions also exhibit loss of at least one copy of a candidate PI3K/mTOR pathway tumor suppressor gene (TSG), *PTEN* (31%), *TSC1* (11%), *TSC2* (13%), *STK11* (34%), and *EIF4EBP1* (36%) [20]. Interestingly, co-occurrence of their gene loss is a highly statistically significant event (Table 1). Similarly, *PIK3CA* amplification co-occurs in a highly statistically significant fashion with gene copy gains in *PI3KCB*, encoding the PI3Kβ subunit. These occurrences of multiple alterations may cooperate to persistently activate PI3K/AKT/mTOR pathway in most HNSCC lesions.

**Table 1:** Frequent co-occurrence of genomic alterations in OSCC. Co-occurrence and mutually exclusivity in genomic alterations in the PI3K/mTOR signaling network (Fig. 1) was computed using the cBioPortal bioinformatics platform. Significant interactions (*p* < 0.05) were included

Gene-gene	*P* value	Log odds ratio	Association
*PIK3CA*-*PIK3CB*	< 0.001	> 3	Co-occurrence
*PTEN*-*TSC2*	< 0.001	0.917	Co-occurrence
*STK11*-*TSC2*	< 0.001	0.889	Co-occurrence
*STK11*-*TSC1*	< 0.001	1.021	Co-occurrence
*PIK3CB*-*PIK3CG*	0.004	1.524	Co-occurrence
*HRAS*-*STK11*	0.017	< −3	Mutual exclusivity
*STK11*-*EIF4EBP1*	0.022	0.403	Co-occurrence
*PIK3CB*-*PTEN*	0.032	0.592	Co-occurrence
*HRAS*-*PTEN*	0.041	< −3	Mutual exclusivity

HPV infection has been recently recognized as a viral etiologic agent responsible for HNSCC, more specifically in the oropharynx [21, 22]. While the overall incidence for HNSCC continues to decrease, it is observed that the incidence of HPV-associated HNSCC has a highly significant increase, predominantly among young patients [23–25]. Among the viral proteins encoded by high-risk HPVs (HPV16 primarily in HNSCC), E6 and E7 function as major driver oncogenic proteins. By disrupting p53 and RB tumor suppressor proteins, respectively [26–28], E6 and E7 induce malignant transformation. Recent findings suggest that HPV+ HNSCC has a significant enrichment of *PIK3CA* mutations (25% more than HPV−) and exhibit elevated mTOR activity [1, 29–31]. Of note, E6 and E7 oncoproteins could not be therapeutically targeted so far, making it essential to explore druggable targets for HPV+ HNSCC, in which mTOR inhibition provides suitable therapeutic options [31].

Taken together, the above findings suggest that, although genomic alterations found in HNSCC varies and are remarkably complex, most fall within certain oncogenic pathways, most of which result in persistent aberrant activation of the mTOR signaling pathway.

## The roles of mTOR signaling pathway in cancer

The mTOR (mechanistic target of rapamycin) pathway regulates major cellular processes involved in organismal growth and homeostasis [32–34]. Dysregulation of this pathway occurs in multiple human diseases, such as cancer, obesity, type II diabetes, and neurodegeneration, to name but a few [33].

In the past decades, mTOR-dependent processes have been continuously uncovered. Briefly, mTOR is an atypical serine/threonine protein kinase. By interacting with several proteins, mTOR encompasses two distinct protein complexes: mTOR complex 1 (mTORC1) (which includes raptor, pras40, deptor, and mLST8) and mTOR complex 2 (mTORC2) (which includes rictor, mSin1, protor1/2, deptor, and mLST8) [33]. Through phosphorylation of two key eukaryotic translation regulators, p70S6K (p70-S6 kinase) and EIF4EBP1 (4EBP1, short for eukaryotic translation initiation factor 4E binding protein 1), mTORC1 regulates ribosomal biogenesis and protein synthesis. In addition, mTORC1 also controls lipid synthesis, autophagy, and metabolism by targeting key effectors SREBP1/2, HIF1α, and ULK1/ATG13/FIP200, respectively [32, 33]. mTORC2 directly phosphorylates AKT at S473, and mTORC2 is required for activation of SGK1, known as serum and glucocorticoid-regulated kinase 1, and plays an essential role in multiple processes including cell survival, neuronal excitability, and renal sodium excretion [35–38]. Collectively, the mTOR pathway regulates cell growth and components of the pathway are key molecules involved in numerous pathological conditions.

Specifically for cancer pathogenesis, many studies have documented the important role of mTOR pathway. Evidence shows that deregulation of protein synthesis controlled by 4E-BP/eIF4E, downstream of mTORC1, plays a central role [39–43]. It is thought that mTOR phosphorylates and represses the inhibitory activity of 4E-BP1 on eIF4E, affecting the translation of mRNA coding for a subset of pro-oncogenic proteins, including cMYC and cyclin D1 [41, 43–49]. Lipid synthesis is characterized as a hallmark for proliferation of cancer cells [50]. SREBP1, a central pro-lipogenic factor, can be activated by mTORC1 [51]. Autophagy has both tumor suppressive and cancer cell survival protective effects. In a nutrient and oxygen deprivation environment, autophagy makes cancer cells insensitive to these stressors and provides survival advantage. Meanwhile, autophagy may cause apoptosis due to lack of energy storage. Activation of mTORC1 signaling inhibits autophagy in cancer cells and may protect against autophagy-induced apoptosis [33, 52]. In addition to mTORC1, recent studies suggest mTORC2 plays a distinct role in multiple cancer types. The mTORC2-AKT-FOXO circuit regulates proliferation, angiogenesis, and apoptosis [53–55].

These evidences point to the importance of mTOR pathway in cancer initiation and progression. Due to the recent characterization of molecular alterations found in HNSCC, we now know that the PI3K/mTOR signaling circuitry is the most frequent dysregulated signaling pathway in HNSCC, as described above. Thus, the use of precise molecular therapeutic approaches to reduce the activity of the mTOR pathway could have anti-cancer effects in HNSCC, and the dissection of the underlying mechanisms may help select the patient population that will benefit the most from this therapy.

## Targeting mTOR signaling pathway in head and neck cancer

Rapamycin, also known as sirolimus, represents the first generation of mTOR inhibitors. It was firstly used as an immunosuppressant since the 1970s. Despite its anti-cancer activity being discovered in the early 1980s, the application of rapamycin for cancer therapy was not exploited until the late 1990s [32]. A class of drugs that target mTOR, termed rapamycin analogues (also known as rapalogs), were subsequently developed [56–58]. Rapamycin and rapalogs block primarily mTOR in its complex 1 (mTORC1) indirectly by binding to FKBP12, while a second generation of mTOR inhibitors block mTOR kinase directly, hence inhibiting both mTORC1 and mTORC2 [34, 57–59].

To investigate the effectiveness of mTOR inhibitors in HNSCC, a series of experimental models has been established. Our group pioneered the use of rapamycin as a single agent to treat HNSCC xenografts; rapamycin rapidly decreased mTOR activity, as indicated by its marker, pS6, and caused rapid tumor regression [60]. Since then, several groups demonstrated anti-cancer effect of rapalogs in HNSCC xenografts either as a single agent or when combined with chemotherapy/radiotherapy [61–66]. Genetically relevant cell lines are necessary for xenograft models, and in recent studies, we reported the detailed characterization of a large panel of HNSCC-derived cell lines by performing exome and transcriptome sequencing [30]. Not surprisingly, genetic alteration in *PIK3CA*, *HRAS*, *PTEN*, and other agents which results in PI3K-mTOR pathway activation were identified in those panels, consistent with the HNSCC genomic landscape described above. These efforts facilitated the identification of biomarkers for diagnosis and treatment, providing selective precision models for xenograft studies [30, 67]. Patient-derived tumorgraft (PDX) models maintain the tumor heterogeneity of the primary tumor; thus, they may be better for clinical outcome prediction. Many recent studies reported the effectiveness of mTOR inhibitors in HNSCC PDX models [68–70]. Meanwhile, chemically induced mouse SCC experimental models were also established. The DMBA-TPA two-stage chemical-induced carcinogenesis is widely used as a mouse SCC experimental model, and in genetically defined animals, such as mice conditionally expressing HPV E6/E7, or lacking Pten or Tgfbr1, DMBA-TPA induces more SCC lesions and more rapidly [71–73]. Another carcinogen, 4-nitroquinoline-1-oxide (4NQO) that mimics tobacco use, was optimized in our laboratory as an oral-specific chemical carcinogenesis model [74]. Using drinking water with soluble 4NQO, oral tumors could be developed in the tongue within several weeks. In both chemical carcinogenesis-induced models, persistent mTOR activation was observed, which could be blocked by administration of rapamycin, hence causing regression of SCCs [71–74]. Overall, these experimental efforts led to a rationale for using mTOR inhibitors as a novel precision therapeutic approach for HNSCC.

Several mTOR inhibitors are being used either alone or in combination with chemotherapy or radiotherapy in HNSCC clinical trials, including rapamycin (sirolimus), everolimus (RAD001), temsirolimus (CCI-779), and others, as summarized in Table 2. Most clinical trials using mTOR inhibitors in HNSCC are under evaluation or have been recently completed. Our study treating newly diagnosed HNSCC patients with rapamycin (NCT01195922) showed encouraging outcomes, as the majority of the patients (15 of 16) gained clinical improvement, including one patient with complete response [75]. Meanwhile, metformin, a drug with well-established safety profile routinely used in type II diabetes patients, has been discovered as a potential anti-cancer agent by reducing mTOR activity in experimental animal models [76, 77]. A multi-institutional phase IIa trial (NCT02581137) in patients with oral premalignant lesion (OPL) has been initiated to evaluate the effect metformin for oral cancer prevention. It is expected that the patients with HNSCC or OPL could benefit from these experimental efforts and relevant clinical trials.

**Table 2:** Clinical trials targeting mTOR in HNSCC. List of clinical trials targeting mTOR in HNSCC or premalignant lesions of the oral cavity, mTOR inhibitors are used either as single agents or in combination with other therapies. *mTORC* mTOR complex, *HNSCC* head and neck squamous cell carcinoma, RT radiation therapy

Identifier no.	Drugs	Target (s)	Combination	Phase	Status	Conditions
NCT01195922	Sirolimus	mTORC1	Single agent	I/II	Completed	Previously untreated HNSCC
NCT02646319	Nanoparticle albumin bound rapamycin	mTORC1	Single agent	III/IV	Recruiting	Advanced cancers with mTOR mutations
NCT01172769	Temsirolimus	mTORC1	Single agent	II	Completed	HNSCC
NCT01015664	Temsirolimus	mTORC1	Cetuximab + cisplatin	I/II	Teminated	Recurrent or metastatic HNSCC
NCT01009203	Temsirolimus	mTORC1	Erlotinib	II	Teminated (has result)	Platinum-Refractory or Ineligible, Advanced
NCT01256385	Temsirolimus	mTORC1	Cetuximab	II	Completed	Recurrent or metastatic HNSCC
NCT00195299	Temsirolimus	mTORC1	Single agent	0	Completed	Newly diagnosed advance HNSCC
NCT01016769	Temsirolimus	mTORC1	Paclitaxel + carboplatin	I/II	Not recruiting	Recurrent or metastatic HNSCC
NCT02215720	Temsirolimus	mTORC1	Cetuximab	I	Recruiting	Patients with advanced or metastatic solid tumors
NCT01058408	Everolimus	mTORC1 and 2	RT + cisplatin	I	Teminated	Locally advanced HNSCC
NCT01133678	Everolimus	mTORC1 and 2	Single agent	II	Not recruiting	Locally advanced HNSCC
NCT01333085	Everolimus	mTORC1 and 2	Carboplatin + paclitaxel	I/II	Completed	Locally advanced inoperableHNSCC
NCT01283334	Everolimus	mTORC1 and 2	Carboplatin + cetuximab	I/II	Completed	Recurrent metastatic HNSCC
NCT01313390	Everolimus	mTORC1 and 2	Docetaxel	I/II	teminated	Locally advanced metastatic HNSCC
NCT01057277	Everolimus	mTORC1 and 2	RT + cisplatin	I	Teminated	Locally advanced inoperable HNSCC
NCT00935961	Everolimus	mTORC1 and 2	Docetaxel + cisplatin	I	Completed	Local-regional advanced HNSCC
NCT00858663	Everolimus	mTORC1 and 2	RT + cisplatin	I	Completed	HNSCC
NCT00942734	Everolimus	mTORC1 and 2	Erlotinib	II	Completed	Recurrent HNSCC
NCT01051791	Everolimus	mTORC1 and 2	Single agent	II	Not recruiting	HNSCC
NCT01111058	Everolimus	mTORC1 and 2	Single agent	II	Not recruiting	Locally advanced HNSCC
NCT01009346	Everolimus	mTORC1 and 2	Cetuximab + cisplatin	I/II	Terminated	Recurrent or Metastatic HNSCC
NCT01332279	Everolimus	mTORC1 and 2	RT + erlotinib	I	Terminated	Recurrent HNSCC with previously RT
NCT01637194	Everolimus	mTORC1 and 2	Cetuximab	I	Completed	Metastatic or recurrent HNSCC
NCT03065387	Everolimus	mTORC1 and 2	Neratinib, palbociclib, trametinib	I	Not recruiting	Advanced cancers
NCT03065062	Gedatolisib	PI3K/mTOR	Palbociclib	I	Recruiting	Advanced HNSCC
NCT01212627	Ridaforolimus	mTORC1 and 2	Cetuximab	I	Terminated	Advanced HNSCC
NCT01353625	CC-115	DNA-PK/TOR	Single agent	I	Not recruiting	Patients with advanced solid tumors
NCT02644122	SF1126	PI3K/mTOR	Single agent	II	Recruiting	HNSCC with PIK3CA or pathway mutations
NCT02402348	Metformin	mTOR *via* AMPK	Single agent	I	Terminated	HNSCC
NCT02949700	Metformin	mTOR *via* AMPK	RT + cisplatin	I/II	Recruiting	HNSCC
NCT01333852	Metformin	mTOR *via* AMPK	Placlitaxel	II	Terminated	Metastatic or recurrent headand neck cancer
NCT02325401	Metformin	mTOR *via* AMPK	RT + cisplatin	I	Recruiting	Locally advanced HNSCC
NCT02083692	Metformin	mTOR *via* AMPK	Single agent	0	Completed	HNSCC
NCT03109873	Metformin	mTOR *via* AMPK	RT	I	Recruiting	HNSCC
NCT02581137	Metformin	mTOR *via* AMPK	Single agent	II	Recruiting	Erythroplakia, oral leukoplakia, OSCC
NCT02917629	Metformin	mTOR *via* AMPK	Single agent	I–IV	Recruiting	Oral cavity or oropharynx cancer

## mTOR co-targeting strategies in HNSCC to bypass drug resistance

Molecular mechanism-based precision medicine provides promising rationale for cancer therapy. However, the clinical efficacy of several agents is frequently compromised due to the emergence of drug resistance. Specifically for therapeutic drugs targeting the mTOR pathway, despite the fact that promising outcomes were achieved using mTOR inhibitors in experimental models and rapamycin showed encouraging result in the adjuvant setting in a HNSCC clinical trial (NCT01195922, Table 2), advanced HNSCC patients may still display unpredictable drug resistance. One possible reason is that HNSCC patients, similar to other cancer types, usually receive multiple rounds of radiation and chemotherapy. This may cause DNA damage; thus, the consequent genetic alterations and epigenetic regulation can induce the emergence of drug resistance.

Understanding the molecular basis of potential drug resistance has emerged to be a formidable challenge. To date, diverse mechanisms of drug resistance have been discovered, including adaptive changes impacting drug pharmacokinetics (such as absorption, distribution, metabolism, and excretion), structural changes in the drug-binding domain of targeted molecules, and (re)activation of pro-survival signaling pathway. Mechanisms of resistance vary depending on the individual drug. For example, metformin, a novel drug candidate for cancer prevention, is proved to inhibit mTOR signaling [78], and low expression of organic cation transporter 3 (OCT3/SLC22A3), a metformin uptake transporter [77, 79] causes resistance to metformin. The immunodetection of OCT3 expression levels in HNSCC cases provide a surrogate marker which may predict a favorable response to metformin, and on the other hand, it may also suggest that patients with low OCT3 expression may be excluded from metformin trials [77, 79].

Changes in the structure of drug target proteins are frequently observed in experimental models when cell lines were continuously exposed to certain precision medicines. Selective pressure leads tumor cells to develop resistant subpopulations. It is reasonable to predict that a patient with long-term use of a drug may experience similar situations. Structure-based functional design of drugs could be used to optimize existing compounds to target altered drug-insensitive proteins. For example, deep sequencing of MCF7 breast cancer cells acquiring mTOR resistance revealed the juxtaposition of the binding sites of rapamycin and AZD8055, an mTOR kinase inhibitor. Based on this knowledge, it was possible to develop a bivalent mTOR inhibitor. This third-generation mTOR inhibitor, named RapaLink-1, maintained activity in both rapamycin-resistant and AZD8055-resistant xenografts in breast cancer [80]. This drug discovery effort provides opportunities for precision medicine approaches to target mTOR for cancer therapy.

Perhaps more often, targeted agents promote the activation of adaptive survival signaling in tumor cells, either by the same or parallel pathways to mTOR. For example, rapamycin-induced feedback phosphorylation and activation of Akt signaling is frequently reported [81–84]. Also, mTOR inhibition by rapamycin and other TOR kinase inhibitors induces tyrosine receptor kinase and ERK/MAPK feedback activation [84–87]. Low expression of 4E binding protein 1 (4EBP1), a primary downstream substrate of mTOR suppressing eukaryotic translation initiation factor 4E (eIF4E), may confer resistance to mTOR inhibitors [39–41]. Other dysregulations include p27, PP2A, PIM, and many others, but their roles in resistance to mTOR inhibition are less characterized [33].

To date, precision therapeutic strategies, mTOR inhibition included, are designed based on the concept of oncogene addiction, in which the growth and survival of cancer cells can be often impaired once one single oncogene is inhibited [88]. However, the discoveries of drug resistance bring to attention the importance of co-targeting strategies. In fact, numerous clinical trials using mTOR inhibitors are being carried out in combination with other targeted therapy (Table 2). The knowledge of the mechanism of resistance to mTOR inhibition may provide optimized second target(s) to be combined with as a therapeutic option. For instance, compensatory increased Akt and/or ERK signaling after mTOR inhibition are responsible for tumor relapse and their targeted agents have been widely investigated [81–87]. Consistent with the above findings, we recently performed a synthetic lethality screen using shRNA libraries in HNSCC cell lines and found that co-targeting numerous molecules involved in ERK/MAPK pathway sensitizes the growth suppressive activities of mTOR inhibition. Indeed, trametinib, a MEK1/2 inhibitor, exhibited a synergistic effect by sensitizing HNSCC to rapamycin [89]. In another study, co-targeting mTOR with cetuximab, a monoclonal antibody targeting EGFR that acts upstream of both Akt and ERK pathway, prevented the growth of HNSCC tumor xenografts by decreasing cell proliferation and lymphangiogenesis [90]. Of importance, safety of cetuximab has been proven and its combination with mTOR inhibitors may minimize their side effects. These pre-clinical efforts have provided a rationale for using mTOR co-targeting strategies in HNSCC patients.

## mTOR and cancer immunology

Cancers, including HNSCC, are immunosuppressive diseases. Cancer cells normally avoid immune surveillance and anti-tumor immune response by recruiting myeloid-derived suppressor cells (MDSC) and suppressive regulatory T cells (Tregs) [91], while macrophages undergo polarization toward an immune suppressive (M2) tumor-associated macrophage (TAM) phenotype [92–94]. In HNSCC, patients often have low absolute lymphocyte counts (ALC), impaired natural killer (NK) cell activity, and poor antigen presentation function compared to healthy volunteers [6]. To recognize cancer cells as foreign instead of self and effectively attack them by the immune system, three general categories, known as immuno-oncology (IO) therapies, could be applied, including checkpoint inhibitors, immune stimulatory cytokines, and cancer vaccines [6, 95]. Recently, revolutionary therapeutic strategies have been used to restore T cell-mediated anti-tumor immunity in HNSCC by targeting immune checkpoint molecules, such a PD-L1 and PD-1. These studies demonstrated immune modulation and durable remissions and led to the recent approval by the FDA of anti-PD-1 antibodies, nivolumab and pembrolizumab, for use in HNSCC treatment [6–11]. Numerous trials in HNSCC are being evaluated. However, the overall response rate to these IO therapies in HNSCC is only ~20% [6–11]. There is a clear need to identify therapeutic options to enhance the response to these IO agents in HNSCC.

Both precision therapies and IO therapies are novel therapeutic modalities being under clinical evaluation for cancer treatment. This provides numerous potential opportunities for synergistic treatment strategies. Specifically for mTOR inhibition, the mTOR pathway was early considered to be a target of immunosuppressive therapy, and rapamycin has been used in renal transplant patients who were also taking cyclosporine and corticosteroids [96]. Nonetheless, rapamycin-treated patients have similar numbers of myeloid dendritic cells (DCs) and plasmacytoid dendritic cells (pDCs), suggesting that at the dose used, rapamycin does not compromise the DC compartments in patients [97]. Moreover, in cancer patients, multiple trials using single-agent rapamycin (or rapalogs) have shown no evidence of increased incidence of immunosuppression [98–100]. Paradoxically, recent basic and clinical studies have associated rapamycin with increased immune responses and potentiation of the activity of IO agents in cancer models [101–109]. Thus, it is possible that co-targeting mTOR may potentially enhance rather than reduce the anti-tumor activity of IO agents.

**Fig. 2:**
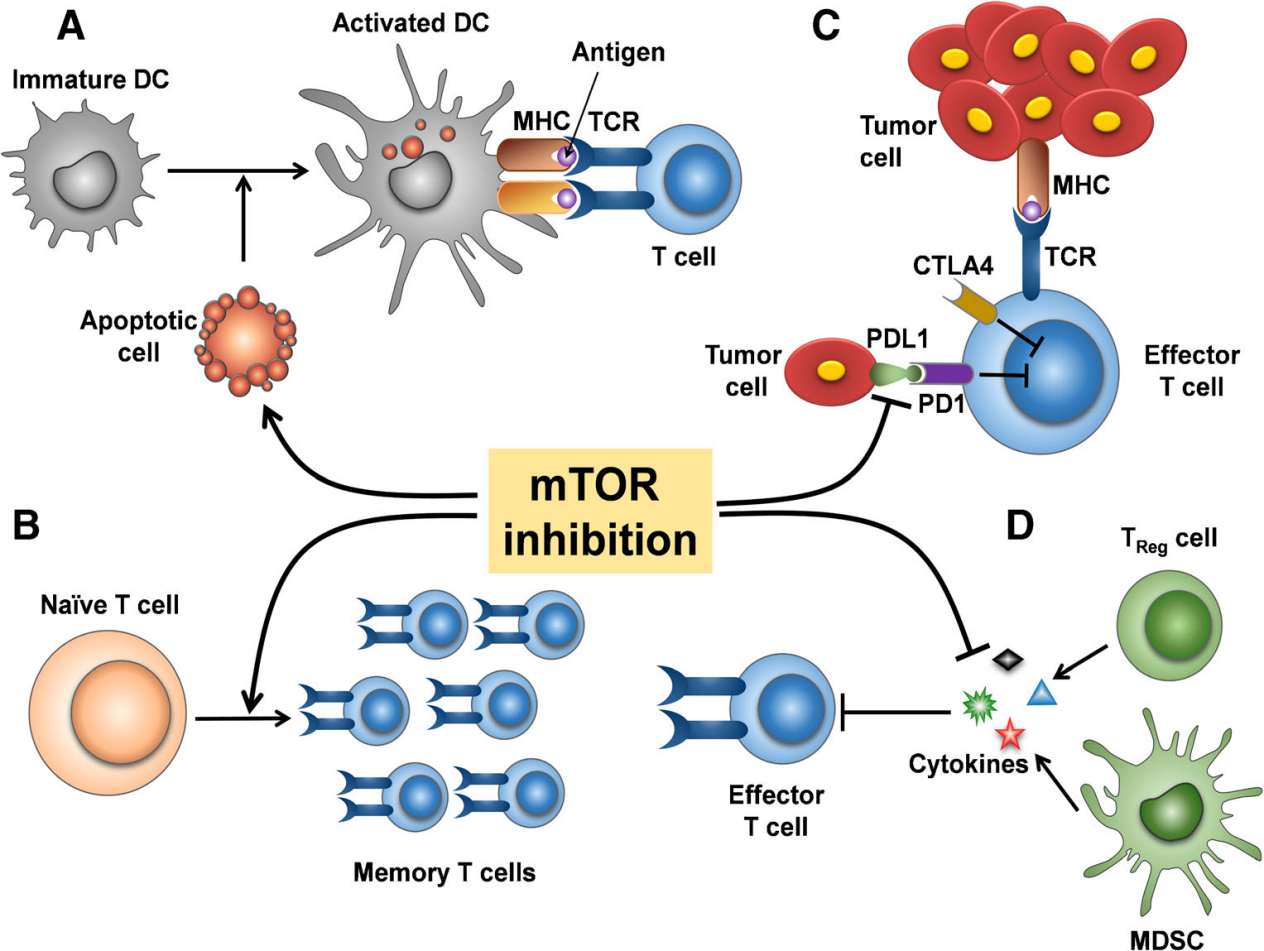
mTOR inhibition may enhance the anti-tumor immune response. A DCs capture tumor antigens and present them to T cells through MHC (class I and class II) pathways. mTOR inhibition induces apoptotic cells, which may contribute as *vaccination in situ*. B mTOR inhibition drives T cells toward long-lived tumor specific memory T cells. C The inhibitory molecule PDL1 from tumor cells can bind PD1 in T cells and weaken effector T cell’s function. Co-targeting mTOR may reduce PD-L1 expression, restraining PD-L1/PD1 mediated inhibition. Effector T cells refer to as cytotoxic T cells (CD8+) and helper T cells (CD4+). D Immunosuppressive cytokines secreted by Tregs and MDSCs inhibit anti-tumor response. mTOR inhibition may prevent cytokine secretion by regulation of their translational control. DCs dendritic cells, MHC major histocompatibility complex, Tregs regulatory T cell, MDSCs myeloid-derived suppressor cells

The generation of anti-tumor immune response usually requires multiple steps, including (1) tumor antigen (peptide epitope) capture, (2) effector T cell differentiation, and (3) evasion of negative regulation [91]. Firstly, mTOR inhibitors, similar to many other cancer precision therapeutic options, may cause programmed cancer cell death [110]. The apoptotic tumor cells killed *in situ* can expose multiple antigens. Similar to a variety of cancer vaccines, those antigens can be processed into major histocompatibility complex (MHC) class I and class II pathways, to activate CD8+ T cells and CD4+ T cells, respectively [91]. Secondly, mTOR has double-edged sword effects during the progress of T cell differentiation. While mTOR activation programs their differentiation into functionally distinct lineages [111], mTOR inhibition drives T cell toward long-lived tumor specific memory T cells [91]. Thirdly, the checkpoint proteins CTLA4 and programmed cell death protein 1 (PD1) are among the major inhibitory molecules suppressing activated T cells [112–115]. We reported in a HNSCC mouse model that an increase in programmed death-ligand 1 (PD-L1) expression can be elicited by *Pten* gene deletion [116], suggesting PI3K/mTOR pathway activation contributes to PD-L1/PD1 stimulation in HNSCC. A recent study showed that co-targeting mTOR and PD-L1 enhances tumor control by increasing the IFNγ production capacity in peripheral and tumor-infiltrating CD8 T cells in a syngeneic oral cavity cancer model [109]. Meanwhile, tolerogenic cytokines secreted by regulatory T (Treg) cells and MDSCs also inhibit anti-tumor immune responses [117, 118]. The expression of inhibitory molecules, such as interleukin-10 (IL-10) and transforming growth factor-β (TGFβ) could be decreased by mTOR inhibition [119–122] (Fig. 2).

Therefore, although counterintuitive, emerging evidence supports that PI3K/mTOR inhibition can be optimized to enhance, rather than suppress, the anti-tumor immune response by overcoming immune evasion in a context-dependent fashion. Thus co-targeting the mTOR signaling circuitry based on the genetic stratification of PI3K/mTOR network subtypes with IO agents, may represent a novel precision immune therapeutic approach for HNSCC.

## Conclusion

In summary, the importance of mTOR signaling circuitry in HNSCC has been well documented, and targeting mTOR as a precision therapy approach in HNSCC has been widely investigated in experimental models, and recently tested in clinical trials. Newly developed genetic approaches could be applied to evaluate the status of mTOR activation which may predict individuals’ clinical response to this precision therapy. This mechanism-based therapeutic approach may help select patient populations that may benefit the most from the concomitant administration of mTOR inhibitors, and also provide improved therapeutic options, namely co-targeting strategies to circumvent innate and acquired resistance to mTOR inhibitors. Furthermore, there is now a strong rationale for co-targeting mTOR with IO agents to enhance their anti-tumor activity. Overall, we can expect that the development of novel mTOR co-targeting strategies may achieve durable responses and cancer remission, hence increasing the life expectancy and quality of life of HNSCC patients.
